# Correction: Conservation of wild food plants from wood uses: evidence supporting the protection hypothesis in Northeastern Brazil

**DOI:** 10.1186/s13002-024-00731-7

**Published:** 2024-09-20

**Authors:** Roberta de Almeida Caetano, Emilly Luize Guedes da Silva, Luis Fernando Colin-Nolasco, Rafael Ricardo Vasconcelos da Silva, Adriana Rosa Carvalho, Patrícia Muniz de Medeiros

**Affiliations:** 1 Postgraduate Program in Biological Diversity and Conservation in the Tropics, Federal University of Alagoas, Avenida Lourival Melo Mota, S/N, Tabuleiro do Martins, Maceió, AL 57072-900 Brazil; 2 Postgraduate Program in Ethnobiology and Nature Conservation (PPGEtno), Federal Rural University of Pernambuco, Rua Dom Manoel de Medeiros, S/N – Dois Irmãos, Recife, PE 52171-900 Brazil; 3 Laboratory of Biocultural Ecology, Conservation and Evolution (LECEB), Campus of Engineering and Agricultural Sciences (CECA), BR-104, Km 85, S/N, Rio Largo, AL 57100-000 Brazil; 4 Department of Ecology, Federal University of Rio Grande Do Norte, University Campus S/N, Lagoa Nova, Natal, RN 59098-970 Brazil

**Correction: Journal of Ethnobiology and Ethnomedicine (2024) 20:81** 10.1186/s13002-024-00719-3

Following publication of the original article, it was brought to the attention of the journal that the name of the second author, Emilly Luize Guedes da Silva, was incorrectly written as ‘Emilly Luize Gomes da Silva’. The error has since been corrected in the original article, and the corrected name may be seen in the author list of this erratum.

